# Resistance to endocrine therapy in breast cancer: molecular mechanisms and future goals

**DOI:** 10.1007/s10549-018-5023-4

**Published:** 2018-11-01

**Authors:** Małgorzata Szostakowska, Alicja Trębińska-Stryjewska, Ewa Anna Grzybowska, Anna Fabisiewicz

**Affiliations:** Department of Molecular and Translational Oncology, The Maria Skłodowska-Curie Institute of Oncology, Roentgena 5, Warsaw, Poland

**Keywords:** Breast cancer, Endocrine therapy, Resistance, Molecular mechanisms

## Abstract

**Introduction:**

The majority of breast cancers (BCs) are characterized by the expression of estrogen receptor alpha (ERα+). ERα acts as ligand-dependent transcription factor for genes associated with cell survival, proliferation, and tumor growth. Thus, blocking the estrogen agonist effect on ERα is the main strategy in the treatment of ERα+ BCs. However, despite the development of targeted anti-estrogen therapies for ER+ BC, around 30–50% of early breast cancer patients will relapse. Acquired resistance to endocrine therapy is a great challenge in ER+ BC patient treatment.

**Discussion:**

Anti-estrogen resistance is a consequence of molecular changes, which allow for tumor growth irrespective of estrogen presence. Those changes may be associated with ERα modifications either at the genetic, regulatory or protein level. Additionally, the activation of alternate growth pathways and/or cell survival mechanisms can lead to estrogen-independence and endocrine resistance.

**Conclusion:**

This comprehensive review summarizes molecular mechanisms associated with resistance to anti-estrogen therapy, focusing on genetic alterations, stress responses, cell survival mechanisms, and cell reprogramming.

## Introduction

Breast cancer (BC) is one of the most common female tumors diagnosed worldwide. In the U.S., BC represents 29% of all new cancer cases in women [1]. Metastatic breast malignancy is also the second most fatal cancer in woman.

In order to choose the most efficient treatment strategy, it is crucial to determine the biological subtype of the examined breast cancer. Biological subtypes are classified according to the expression of steroid receptors (estrogen (ER) and progesterone (PR)) and HER-2 (human epidermal growth factor receptor 2) established via immunohistochemistry (IHC) (Table 1). Cancers that are positive for estrogen receptor (ER+) and/or progesterone receptor (PR+) in IHC are classified as luminal. Currently, more accurate molecular expression profiling is available (PAM50 assay) that enables to distinguish more accurate risk groups (high recurrence risk subtypes) [2–4].

**Table 1:** Biological subtypes of breast cancer distinguished by IHC

Biological subtype	ER	PR	HER-2	Recommended I line treatment	Frequency	References
Luminal A	+	+/−	−	Endocrine therapy	40–50%	[5–7]
	(−)	(+)			(< 3%)	
Luminal B	+	+/−	+/−	Endocrine therapy combined with chemotherapy	20–30%	[5, 7]
	(−)	(+)			(< 3%)	
HER-2 enriched	−	−	+	Anti-HER2, adjuvant therapy, chemotherapy	20–30%	[5, 8]
Basal-like (Triple-negative)	−	−	−	Systemic chemotherapy	~ 15%	[5, 9]

Luminal cancers have a better prognosis than other types of BC and are sensitive to anti-estrogen therapy [10, 11]. They are further divided into subgroups (luminal A, B and C), which differ in aggressiveness. Luminal A breast cancers are characterized by high expression of luminal epithelial genes, low expression of Ki-67 and distinct methylation profile of more than 40 gens (see review: [12]). The luminal B breast cancers are characterized by higher Ki-67 and lower expression of several luminal-related genes (like *ESR1* or *FOXA1*), genomic instability and a higher frequency of *TP53* gene mutations; thus, they are associated with a worse prognosis and a higher risk of relapse than luminal A breast cancers [5, 12]. Luminal C subtype, characterized by molecular profiling and unrecognizable via IHC displays the overexpression of genes that are characteristic for non-luminal breast cancers, like transferrin receptor (CD71), *MYB*, nuclear protein P40, *SQLE*, and *GGH* [5].

Luminal breast cancers constitute the majority of diagnosed breast cancers (Table 1) [5]. Non-luminal breast cancers (HER2 positive, triple-negative breast cancer) have poorer prognosis than luminal cancers. HER2-positive cancers represent 20–30% (Table 1) of all diagnosed breast cancers and can be treated with anti-HER2 antibodies [5, 8]. Triple-negative breast cancer (TNBC; without ER, PR and HER2 expression) represents nearly 15% (Table 1) of BCs and have short disease-specific survival and poor prognosis [5, 13].

The “gold standard” treatment of luminal breast cancer is anti-estrogen therapy. The aim of this treatment is to block the effect of estrogen at the receptor level (selective estrogen receptor modulators, SERM/selective estrogen receptor down-regulators, SERD) or by inhibiting estrogen production (aromatase inhibitors) [14–16].

Despite the high sensitivity of luminal tumors to endocrine therapy, 30–50% of early breast cancer patients will later relapse. Additionally, these cancers have a tendency to stay dormant, often for many years and metastasis can be triggered as late as 20 years after diagnosis [6, 17]. Resistance to therapy and distant metastases are the main causes of death in breast cancer patients [10].

## Estrogen receptors

There are three major forms of physiological estrogens in females: estrone (E1), estradiol (E2, or 17β-estradiol), and estriol (E3) [18]. The estrogen receptor acts as a ligand-dependent transcription factor and has two forms: ERα and ERβ, encoded by the *ESR1* and *ESR2* genes, respectively [19]. ERα is a transcription factor for genes associated with cell survival, proliferation, and tumor growth (e.g., genes for insulin-like growth factor-1 receptor (IGF1R), cyclin D1, anti-apoptotic BCL-2 protein, vascular endothelial growth factor (VEGF)) [20]. The phosphorylation of ERα has a profound impact on its activity and ERα-regulated gene expression, as well as on cell growth, migration, and morphology [21]. While the role of ERα in tumorigenesis is crucial, the role of ERβ is still controversial [22]. It is believed that ERβ has anti-proliferative properties; thus, it acts as an ERα antagonist and is not expressed in breast cancer cell lines [23]. However, detailed validation of commonly used ERβ antibodies has demonstrated that some of these reagents either detect ERβ only in specific experimental conditions or lack any specificity for ERβ across multiple assays. Therefore, our current understanding of ERβ role in cancer may be not accurate [24] and should be revalidated. The newest study on ERβ + TNBC cell lines suggests that ERβ expression may be the prognostic factor for TNBC patients. In ERβ+ cells, E2 is an activator for ERβ signaling, which induces cystatins and results in inhibition of TGF-β signaling pathway. This suppression results in decrease of TNBC cells invasiveness in vitro [25].

Both ERα and ERβ possess several functional domains: the N-terminal domain (NTD), the DNA binding domain (DBD), and the ligand binding domain (LBD) [26]. Moreover, there are two activation domains (AFs) that are part of the NTD and LBD functional domains (AF1 and AF2, respectively). Activation domains in the agonist conformation are involved in the recruitment of co-activators and co-repressors for certain target genes.

**Fig. 1:**
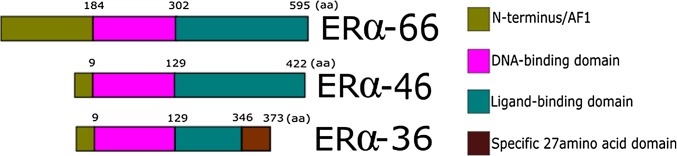
Scheme of ERα, ERα-46, and ERα-36 structure

Full length estrogen receptor alpha has 66-kDa (ERα-66), but has a few isoforms coded by alternatively spliced mRNAs, from which best known are ERα-46 and ERα-36 [27] (Fig. 1). ERα-46 was found in over 70% of breast tumors and displayed variable expression levels, sometimes higher than ERα-66. In this isoform alternative splicing results in the removal of N-terminal AF1 transactivation domain [28]. If ERα-66 and ERα-46 are co-expressed, ERα-46 acts as a competitive inhibitor of ERα AF1 [29]. ERα-36 lacks both activation factor sequences (AF1, AF2), but the DNA-binding domain, part of the ligand-binding domain and the propensity for partial dimerization are retained in this variant; thus, it may recognize the ligand. It is hypothesized that ERα-36 may primarily function as a membrane-based estrogen receptor to mediate membrane-initiated, non-genomic, estrogen signaling [27]. It was also shown that tamoxifen acts as an agonist on ERα-36 in breast cancer cells, promoting stemness and contributing to hormone therapy resistance and metastasis [30]. However, there are still no clinical data from trials taking into account ERα-36 expression in BC patients.

## Resistance to endocrine therapy

In 15–20% of cases, the resistance is associated with the activation of an ERα-independent proliferation mechanism. It may be accompanied by a phenotypic change in cells, from ERα + to ERα−. As luminal B breast cancers are characterized by the lower expression of ERα than luminal A breast cancers, there is a higher probability of ERα expression loss in luminal B breast cancers [5, 31]. Therefore, luminal B breast cancers are considered to have a higher risk of acquiring endocrine resistance than luminal A breast cancers.

This review will focus on molecular mechanisms of estrogen-independence, ER switch and the role of miRNA, autophagy and stemness in acquiring resistance to endocrine therapy.

### *ESR1* modifications

As ERα (in this review referred to as ‘ER’) is the main target in anti-estrogen therapy, changes in *ESR1* gene may lead to estrogen-independence [32].

#### Mutations

**Fig. 2:**
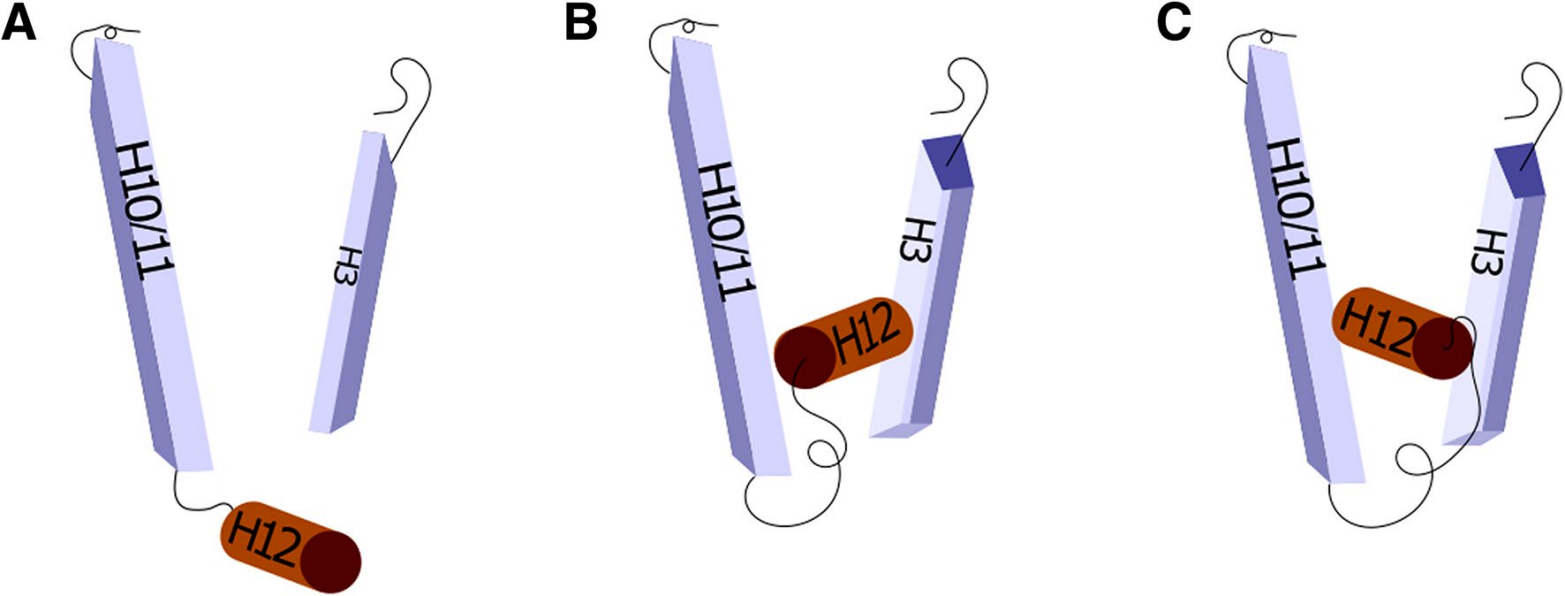
Structure and conformation of helix 12 (H12) of the ER LBD. Scheme shows a fragment of the full ER protein and rollers stand for α-helix structures. **a** Position of H12 when E2 or SERD/SERM is not bound to ER; **b** Position of H12 in antagonist ER conformation; **c** Position of H12 in agonist ER conformation. Missense mutations cause conformational changes placing H12 in position of agonist conformation. Place and amino acid of substitution determines bond straight and stability of H12 position

One of the activation domains of estrogen receptor (AF2) is located at the end of the ligand-binding domain (LBD) and consists of four helices (H9–H12). Those helices are flexible, and their conformation is dependent on the ligand [33]. The crucial structural element for estrogen-dependent AF2 activity is an α-helix, named ‘helix 12’ (H12). When LBD is bound to the E2, helix 12 unveils the binding place for ER co-activators. However, when bound with SERM or SERD, helix 12 changes its orientation and covers the binding place for ER co-activators [34, 35] (Fig. 2). The length of helix 12 is different for agonist- and antagonist-bound structures; agonist-bound structures start at D538 and antagonist-bound structures at L536 [34]. Substitutions of amino acids: L536, Y537, D538 result in conformational changes, placing H12 in the agonist position. This leads to constitutive transactivation activity in the absence of a ligand. The stability and activity of these mutants are dependent on H11–H12 interaction. Strength of this interaction depends on the amino acid that replaced wild-type occurring amino acid [34–37]. E380Q, another substitution associated with H12 positioning, is placed away from H12 sequence, but is close to the carboxy-terminal portion of H12. In wild-type, glutamic acid (E) induces the repulsion between H5 and H12 that unfavorites the generation of agonist H12 position. Substitution to glutamine (Q) abolishes that repulsion, which allows H12 to adopt the agonist position without the E2 [38].

The point missense mutations in *ESR1* gene (Table 2) are associated with the constant ER activity. However, the clinical value of those single nucleotide variations (SNVs) remains debatable. The *ESR1* mutations are infrequent in the primary tumor, but their frequency in metastatic lesion may be even 30-times higher [39]. Meta-analysis of the clinical value of *ESR1* mutations detected in cfDNA (cell-free DNA) revealed that they may have prognostic significance and clinical value in guiding further endocrine therapy choice in ER + MBC patients who received prior AIs therapy, but not for patients treated with fulvestrant [40].

**Table 2:** Most common missense mutations in *ESR1* gene

*ESR1* point mutation	Substitution	Frequency in BC patients (%)	References
1609 T>A	Y537N (Tyr537Asn)	5–33	[37, 41]
1610 A>C	Y537S (Tyr537Ser)	13–22	[36, 41]
1613 A>G	D538G (Asp538Gly)	14–36	[41–43]
1608 TC>AG	L536Q (Leu536Gln)	< 5	[41]
1138G>C	E380Q (Glu380Gln)	< 7	[43]

#### Translocation

Recent studies on breast cancer cell lines and patient-derived xenografts revealed a resistance-associated translocation of *ESR1, ESR1*/*YAP1* (t(6;11)) [44, 45]. The break point of *ESR1* is located at the beginning of helix 12. The fusion protein consists of the N-terminal and DNA binding domains of ER and the C-terminus from YAP1 [44]. YAP1 is the nuclear effector in the HIPPO pathway, which is involved in cell development and growth. *YAP1* is one of the crucial genes in the regulation of proliferation, apoptosis, and stemness. Moreover, overexpression of this gene in breast cancer is associated with the promotion of proliferation, invasiveness, and cell survival [46]. Functional properties of the fusion protein include the following: estrogen-independent growth, constitutive expression of ER target genes, anti-estrogen resistance, activation of a metastasis-associated transcriptional program, and inducing the cellular motility [47]. Newest studies identified multiple 3′ partners for *ESR1* rearrangement, including *SOX9, DAB2, GYG1, MTHFD1L*, and *PLEKHG1, CCDC170* [48, 49]. The cluster junctions for *ESR1* remain similar to the *ESR1*/*YAP* translocation, placed between exons 6 and 7. Activity of fusion protein is independent on the estrogen presence, but its level seems to be dependent on 3′ partner. For example, fusion with *SOX9* or *DAB2* results in 40 times higher activity of fusion protein than wild-type ER, while fusion with *GYG1* results in 2–3 times higher activity than wild-type ER. While it is undoubtable that *ESR1* fusions are demonstrating constitutive, ligand-independent activity [49], there is still lack of evidence for clinical utility of described *ESR1* alterations.

#### Amplification

The amplification of *ESR1* was widely studied [50–56]; nonetheless there are still controversial data about its frequency and prognostic value. Amplification frequency detected in breast cancer patients varies from 1 to 37% [50–54, 56]. The association of *ESR1* amplification and anti-estrogen resistance is also discussed. Some studies report that *ESR1* amplification correlates with tamoxifen resistance and shorter disease-free survival [55, 57], while other studies showed no correlation [54, 58].

### miRNAs as regulators of resistance to endocrine therapy

The up-regulation of some miRNAs can be associated with anti-estrogen resistance, either mediated by the activation of alternate growth pathways or by the inhibition of ER expression. miR-155, miR-221/222, miR-21, miR-125b are assumed to be key upregulated miRNAs in a breast cancer resistance to different types of treatment (see review: [59]). Yet miR-155 and miR-221/222 seem to be most associated with resistance to antiestrogen therapy. miR-155 targets SOC6 (inhibitor cytokine signaling) and stimulates the activation of STAT3 signaling pathway. This pathway is associated with cell survival and resistance to tamoxifen [59, 60]. miR-221 and miR-222 were found to regulate various oncogenic pathways and their high expression was found to be highly correlated with tamoxifen resistance [61, 62]. MCF-7 cells transfected with miR-221/222 overexpress components of TGF-β and TP53 signaling pathways. Also, these miRNAs up-regulate the transcriptional activity of β-catenin and the expression of the Wnt pathway proteins: WNT5A and FZD5. At the same time, miR-221/222 inhibit the expression of P27 and Wnt pathway inhibitors: AXIN2, SFRP2, CHD8, and NLK [61–63]. The role of Wnt pathway deregulation in cancer development is well known (reviewed: [64, 65]). P27 is cycle-dependent kinase inhibitor (CDKI) from the CIP/KIP family of CDKIs. The inhibition of P27 drives cell proliferation by relieving G1 arrest [66]. This deregulation of the cell cycle is found in ER+ breast cancer cell lines resistant to tamoxifen [61, 62]. The expression level of P27 was found to be predictive for patients treated with tamoxifen [67]. Furthermore, exosomes with miR-221/222 can be transferred from resistant cells to sensitive cells, promoting tamoxifen resistance [61].

The down-regulation of some microRNAs can also be associated with endocrine resistance. The low expression of the ERα-36 inhibitors miR-210 and let-7 [68, 69] leads to overexpression of this isoform and ERα-36-mediated anti-estrogen resistance. Let-7 miRNAs match the 3421 to 3442 region located in the 3′UTR of the ERα-36 coding transcript, inducing mRNA degradation and ERα-36 down-regulation [69]. ERα-36 was found to be an ERβ antagonist and agonist of estrogen-like SERM effects [70]. The high expression of this ER isoform is observed in TNBC and luminal cancers resistant to endocrine therapy with an ER expression switch [71]. This may suggest that the loss of ER expression in luminal BCs could be associated with the increased production of ERα-36. In vitro studies confirmed that the low expression of let-7 miRNAs results in ERα-36-mediated endocrine resistance, whereas the restoration of let-7 miRNAs expression leads to a loss of tamoxifen resistance in the MCF-7 tamoxifen-resistant cell line [69]. Another miRNA, miR-873 is involved in the phosphorylation and activation of ER. A direct target of miR-873 is cycle-dependent kinase 3 (CDK3), which phosphorylates ER at Ser104, 106, and 118, leading to enhanced transcriptional activity of ER. Overexpression of CDK3 in breast cancer is associated with increased cell proliferation and resistance to endocrine therapy. The restoration of miR-873 expression results in the decreased activity of ER and sensitizes breast cancer cells to tamoxifen in vivo [68]. Due to the effect of restoring abovementioned miRNAs expression on tamoxifen resistance, they are considered to be used in future target therapies for patients with resistant breast cancers.

### Unfolded protein response (UPR)

Unfolded protein response (UPR) is a sensor system for endoplasmic reticulum (EnR) stress caused by the accumulation of unfolded or misfolded proteins. UPR signaling was show to regulate both autophagy and apoptosis [72]. There are three main arms of UPR: (1) autophosphorylation of protein kinase RNA-like endoplasmic reticulum kinase (PERK), which leads to transient inhibition of protein synthesis; (2) proteolytic cleavage and activation of transcription factor ATF6α and (3) oligomerization and autophosphorylation of inositol-requiring enzyme 1 (IRE1α), which controls alternative splicing of XBP1 mRNA (X-box binding protein 1). Spliced variant of XBP1 may act as transcriptional activator for BCL2 [73], which is known as an anti-apoptotic protein and one of the autophagy suppressors [74]. UPR is sensitive to intracellular calcium concentration: in the absence of stress, the three UPR molecular sensors are maintained inactive through the association with calcium-dependent chaperone; depleted calcium stores in endoplasmic reticulum cause the removal of this chaperone from sensors, leading to UPR activation. Subsequent steps include induction of protein-folding chaperones, resulting in increased folding and protein degradation.

Two different modes of UPR were described: “reactive”—classic response to EnR stress and “anticipatory”—a hormone-dependent pathway in which cells mildly pre-activate the UPR [75]. Mild activation of UPR results in enhanced resistance to stress, representing survival advantage, while strong, sustained activation leads to cell death. It has been shown that hormonal activation of UPR occurs without accumulation of unfolded proteins and involves opening of EnR calcium channels. Among UPR-activating steroid hormones, E2 is prominent. There is a strong correlation between the expression of UPR gene signature and the expression of estrogen regulated genes [76]. Moreover, a strong correlation of UPR gene signature with subsequent resistance to tamoxifen therapy was observed in ER+ breast cancer patients [76]. It was demonstrated that either E2 or competitor antiestrogens and aromatase inhibitors activate mild, pro-survival UPR [77].

### Autophagy

Autophagy is an essential process for homeostasis and the survival of eukaryotic cells. This process eliminates damaged or old organelles, and removes long-lived proteins and/or protein aggregates; thus, it is responsible for the quality control of essential cellular components [78]. Mechanisms of autophagy regulation are well known (reviewed: [79]) and involve unfolded protein response (UPR), phosphatidylinositol 3-kinase (PtdIns3K, PI3K), mammalian target of rapamycin (mTOR) and glucose-dependent protein 78 (GPR78) [80].

In breast cancer cells, autophagy was identified as an initiator of activated cell death induced by anti-estrogen drugs (ACD II) [81]. However, recent studies have recognized autophagy as a survival mechanism. While ACD II is associated with endoplasmic reticulum stress, autophagy allows cells to survive under this condition [78]. Although the increased activation of autophagy is found in cells after anti-estrogen treatment, only 20–30% of MCF-7 population undergo apoptosis [82]. The detailed mechanism of autophagy-mediated resistance is still not clear, there is a strong evidence that the inhibition of autophagy increases sensitivity of cancer cells to anti-estrogen treatment [82–84]. In vivo models showed that autophagy inhibitors restore antiestrogen sensitivity in resistant tumors [85].

#### Mammalian target of rapamycin (mTOR)

**Fig. 3:**
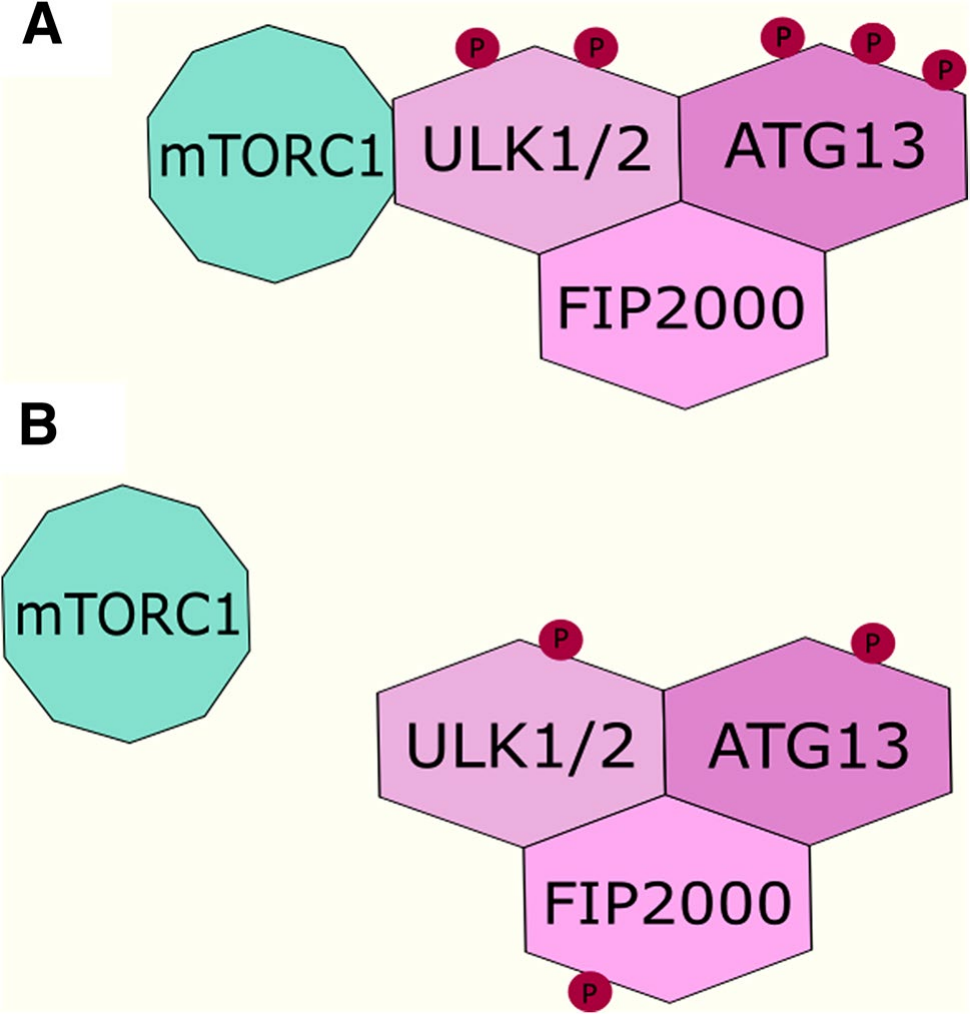
Scheme of regulation of ULK1/2-ATG13-FIP200 complex by mTORC1. **a** mTORC1 phosphorylates ULK1/2 and ATG13 in nutrient-rich conditions; formation of the autophagosomes is suppressed. **b** mTORC1 detaches from complex; ULK1/2 and ATG13 are partially dephosphorylated; induced formation of autophagosomes

Mammalian target of rapamycin (mTOR) kinase is the component of two mTOR complexes: mTORC1 and mTORC2. mTORC1 is a major downstream target of the PI3K/AKT pathway and a negative regulator of autophagy. mTORC1 complex phosphorylates ULK1/2 and ATG13 in nutrient-rich conditions, leading to the deactivation of these complexes. In starvation and stress conditions, the expression of mTORC1 is decreased, and ULK1/2 and ATG13 complexes are partially dephosphorylated. This process activates these proteins and enables the formation of a ULK1-ATG13-FIP200 complex, which is essential for autophagosome formation [86] (Fig. 3). The down-regulation of mTORC1 by rapamycin or PI3K/AKT signaling inhibition leads to an increase in autophagy and can support the acquisition of resistance to tamoxifen in breast cancer cells. Despite the insulin/growth factor and PI3K/AKT pathway being a negative regulators of autophagy, their hyperactivation is well characterized as an anti-estrogen resistance mechanism (reviewed: [87, 88]). Clinical trials with the use of PI3K/AKT inhibitors combined with aromatase inhibitors revealed that PI3K/AKT inhibitors were efficient only in patients with identified PI3K/AKT hyperactivation [89]. In fact, those inhibitors combined with endocrine therapy may be detrimental for some patients. Activation of the PI3K/AKT pathway in stroma is crucial for stroma-associated tumor regression. An in vivo study highlighted that in cancers with low basal expression of PI3K pathway components, treatment with PI3K/AKT inhibitors impaired this process [90].

### Cancer stem-like cells

Cancer stem-like cells (CSCs) are a small population of tumor cells that are capable of self-renewal, producing heterogeneous lineages of cancer cells that comprise the tumor. CSCs are a unique biological subpopulation of cancer cells that could survive indeterminately [91]. Those cells are characterized by the expression of gene sets associated with embryonic stem cells, the activation of multidrug resistance (MDR) and/or DNA-damage response (DDR) systems [92, 93].

Although detailed processes of the formation of cancer stem-like cells are still under debate, it is certain that cancer cells must go through the process of cell reprogramming. This process resets differentiated cells to a pluripotent state and can be achieved by nuclear transfer, cell fusion and/or overexpression of certain transcription factors: *Oct-4, Sox2, Klf4, c-Myc* (OSKM) [94] (human homologs: *POU5F1, SOX2, KLF4, MYC*). As CSCs overexpress *SOX2, POU5F1*, and *NANOG*, it is suspected that reprogramming of cancer cells is mediated via overexpression of those transcription factors [95]. From stemness markers, *SOX2* is best known for its association with anti-estrogen resistance. In vitro studies showed that in ER− endocrine resistant MCF-7 cells SOX2 expression is significantly higher than in ER+-resistant MCF-7 cells [95]. Other in vitro studies revealed that in ER+ endocrine resistant cell lines, SOX2 expression level is negatively correlated with ER and PR expression level. Interestingly, the high expression of SOX2 was also correlated with increasing histopathological grade during tamoxifen resistance acquisition [96]. There is growing evidence that *SOX2* expression is highly correlated with resistance mechanisms and epithelial-mesenchymal transition (EMT)-specific gene expression in cancers. *SOX2* is the regulator of: GLI1, FOXA1, mTOR, EGFR, and WNT and/or NF-κB pathway genes expression [95–100]. Those pathways are associated with growth, proliferation, dedifferentiation and resistance in cancer cells. Moreover, overexpression of other stemness markers can lead to hyperactivation of migration, survival and proliferation associated pathways: HEDGEHOG, WNT, NF-κB, TGF-β, NOTCH, ERK/MAPK (reviewed: [64, 101–103]). Activation of these pathways can lead to the acquisition of anti-estrogen resistance, increased invasiveness, migration and formation of distant metastases.

## Summary and future goals

As described in this review, there are multiple anti-estrogen resistance mechanisms in breast cancer cells, which pose a great challenge in BC treatment. It seems plausible that some of those resistance pathways and systems are linked, which must be taken into account when designing new treatments. For example, PI3K inhibitors impair the stroma-mediated tumor regression of BC in mouse models [90]. Moreover, these inhibitors can promote the activation of cell survival mechanisms related to endocrine resistance, like autophagy. There is a need for a better understanding of the plausible cross-regulation of different resistance-associated molecular mechanisms, to improve the development of targeted drugs. Furthermore, it is essential to validate the detection techniques, prognostic and value of molecular mechanisms described above.
